# Notes on "Some Dreams and Their Significance"
1Read at the Meeting of the Local Branch of the Medico-Psychological Association, held at Brislington House, April 18th, 1912.


**Published:** 1912-06

**Authors:** George H. Savage


### NOTES ON "SOME DREAMS AND THEIR SIGNIFICANCE."[1]

BY

SIR GEORGE H. SAVAGE, M.D., F.R.C.P.

---

I HAVE promised to read a paper at this your local meeting, and I will keep my promise, though I feel more practical gain would follow investigation of the actual treatment of mental disorders at Brislington House. The subject I selected was "Some Dreams and their Significance." Many of you know

[1] Read at the Meeting of the Local Branch of the Medico-Psychological Association, held at Brislington House, April 18th, 1912.

already that I am a very experienced student of dreams. I hardly ever close my eyes without dreaming, and it has therefore been a subject of interest with me to trace the nature of dreams in the sane and in the insane, to see if they can assist us in our diagnosis or treatment.

First, I must give some sort of definition of a dream. I prefer this one: "Mental activities which occurring during sleep are more or less recalled on awaking." Everyone tells you that the lower animals dream, and refer at once to their dog, who moves his feet as if pursuing when asleep on the hearth-rug. That mental action is going on during sleep one admits, but we cannot tell if the dog has any memory of this on waking. I take it that we all believe that mental action of some sort is as constant as heart's action, and continues until death. Most of us must have seen evidences of this in automatic acts performed near the point of death, but a dream rises to a point of consciousness.

To proceed with the subject, dreams may assist in diagnosis of mental disorder. Since the classical description of the epileptic old lady, who always saw someone approaching her to knock her down with a crutch before a fit, we all recognise that dream aura may precede a fit. I have met with cases in which there was always the same dream, and this dream may be followed by automatic action with complete mental oblivion. Such cases provide some of the persons who are picked up wandering in the streets, and though rare, yet it is established I believe that criminal acts depending on a dream may follow during the automatic stage. I have met with certain insane persons who have had definite hallucinations following dream impressions. I fancy many of us know the hypnotic hallucinations which arise on awakening and the persistence of a kind of mental after-image. I have frequently talked to persons who have been recovering from delirium, and they have asked whether such and such a thing has taken place, only to learn that the idea was a mistake. One I remember well, a medical friend who was much puzzled at the confusion which arose in his mind from the recalling of dream memories and others which were of events which really occurred, as he was passing from a delirious to a normal state. I am convinced that some cases about which I have been consulted, in which there have been accusations of indecent assault or even rape, depended upon erotic dreams occurring in adolescents. Dreams occurring in adolescence or at critical periods may lead to various delusions. I have traced a number of sexual delusions and ideas of moral fault to dreams which have been unusually vivid in middle-aged women. The origin and development may be in this way. A woman, often a widow or middle-aged spinster, has one or more very distressing dreams with sense of full sexual gratification. This has given rise in some to the feeling that the ideas are evil, and that they are in the hands of the Devil. In one very distressing case a lady of high birth and of very narrow religious views, after such a dream, was persuaded that the Devil, in whom she fully believed, had during the night visited her, and that she was pregnant of a devil. It is hardly necessary to do more than to point out how such dreams may affect an unstable mind.

I have seen several elderly men who had arterial degeneration who had very disturbing dreams with persistent hypnotic hallucinations. Thus in one case the patient would wake with a dream that persons were in his room making use of his toilet things, and he would have the vision so clearly before his eyes on waking that he would jump up to drive them away. These and similar dream started fancies passed into true visual and lasting hallucinations.

I now pass to the dreams which have been of the same type, but which have preceded attacks of insanity not allied to epilepsy. One lady, whose case I have described elsewhere, had after a good deal of domestic strain a vivid dream that she saw her brother with his throat cut, and that she felt in some way she had been the cause of this. This dream was followed almost immediately by an attack of profound melancholia, in which the dominant idea was that she had caused endless trouble and disgrace to her family. She recovered, and some years later had a similar dream, followed by a like attack of melancholia, with the old delusions. A third dream, followed by a third mental breakdown, followed; but the last attack was slight, as the patient had learnt her lesson, and after the dream at once placed herself under treatment. I may say that this lady, having passed middle life, has now for many years been perfectly stable. I have seen several instances in which an attack of mania has followed a dream of horror or terror.

Next, as to the dreams of the insane. Many insane patients never complain of dreams ; many, in a state of exaltation, are not trustworthy. I have heard marvellous tales which I have not been able to accept, but from what I have been able to gather the maniacal patient's dreams are of the confused and delirious type, not much more incoherent than those of the ordinary dreamer ; but I have met several instances in which men who have had previous attacks of mania have been warned by certain dreams that an illness was impending. These are like some of the so-called prophetic dreams, in which persons have dreamt that they had some lung trouble, and pneumonia has developed in a few days. In melancholic patients the dreams are miserable, and are but part of the morbid feeling, but when they begin to improve the dream-life is more happy. In fact, I never fail to ask about the dreams of the melancholics, for I always expect to hear of peace and happiness coming first during sleep. I think I may say that I never knew a melancholic patient who had begun to dream happily who did not recover. Such persons often say they wish they did not wake at all, as they are so happy in their dreams. The same thing may occur in mania, but this is more difficult to gauge. I have known a maniacal patient who has become quiet, and when spoken to has said he dreamt of home and felt more contented.

And now to sum up. Dreams are thought by some to be reversions to primitive thought ; insanity has been said to be a waking dream, and dream a sleeping insanity. Hughlings Jackson said, " Find out all about dreams, and you will know all about insanity." These general aphorisms I leave with you for your future digestion.

